# Surgical drainage after limb salvage surgery and endoprosthetic reconstruction: is 30 mL/day critical?

**DOI:** 10.1186/s13018-021-02276-x

**Published:** 2021-02-15

**Authors:** Jiayong Liu, Zhengfu Fan, Marc El Beaino, Valerae O. Lewis, Bryan S. Moon, Robert L. Satcher, Justin E. Bird, Spencer J. Frink, Patrick P. Lin

**Affiliations:** 1 Present address: Department of Bone and Soft Tissue Tumor, Peking University Cancer Hospital and Institute, Beijing, China; 2 Present address: Department of Orthopaedic Surgery and Rehabilitation Medicine, State University of New York, Downstate Health Sciences University, Brooklyn, NY USA; 3 Department of Orthopedic Oncology, MD Anderson Cancer Center, Houston, TX USA

**Keywords:** Periprosthetic infection, Surgical drain, Sarcoma, Bone resection

## Abstract

**Background:**

Periprosthetic infection is a major cause of failure after segmental endoprosthetic reconstruction. The purpose of this study is to determine whether certain aspects of drain output affect infection risk, particularly the 30 mL/day criterion for removal.

**Methods:**

Two hundred and ninety-five patients underwent segmental bone resection and lower limb endoprosthetic reconstruction at one institution. Data on surgical drain management and occurrence of infection were obtained from a retrospective review of patients’ charts and radiographs. Univariate and multivariate Cox regression analyses were performed to identify factors associated with infection.

**Results:**

Thirty-one of 295 patients (10.5%) developed infection at a median time of 13 months (range 1–108 months). *Staphylococcus aureus* was the most common organism and was responsible for the majority of cases developing within 1 year of surgery. Mean output at the time of drain removal was 72 mL/day. Ten of 88 patients (11.3%) with ≤ 30 mL/day drainage and 21 of 207 patients (10.1%) with > 30 mL/day drainage developed infection (*p* = 0.84). In multivariate analysis, independent predictive factors for infection included sarcoma diagnosis (HR 4.13, 95% CI 1.4–12.2, *p* = 0.01) and preoperative chemotherapy (HR 3.29, 95% CI 1.1–9.6, *p* = 0.03).

**Conclusion:**

Waiting until drain output is < 30 mL/day before drain removal is not associated with decreased risk of infection for segmental endoprostheses of the lower limb after tumor resection. Sarcoma diagnosis and preoperative chemotherapy were independent predictors of infection.

## Background

Periprosthetic infection is a potentially devastating complication for oncologic patients undergoing segmental bone resection and endoprosthetic reconstruction. Even after multiple operations and extended courses of antibiotics, infection can ultimately be the cause for loss of limb rather than the original neoplasm. With an incidence of approximately 10% for large segmental prostheses, periprosthetic infection represents a major mode of failure [1–3].

There are many serious consequences of infection [4, 5]. Hospitalization may be prolonged, and the cost of treatment can be substantial [6, 7]. Function can be compromised as a result of joint stiffness, increased pain, and, for some patients, amputation [8–11]. A delay in resumption of adjuvant chemotherapy for sarcomas could jeopardize survival [12].

The surgical drain poses a potential risk for infection because it can create a conduit to the skin and become colonized with bacteria [4]. Persistent postoperative drainage beyond 48 h has been reported to be a risk factor for infection after conventional arthroplasty [4, 13–15]. Postoperative drainage is greater in patients undergoing segmental endoprosthetic reconstruction than those undergoing primary joint arthroplasty since a much larger volume of tissue must be removed. This may be one reason why the rate of periprosthetic infection is higher after modular segmental endoprosthetic reconstruction compared to conventional arthroplasty [8]. However, there is little data to guide surgeons in the management of surgical drains after tumor resection. It is still unknown whether multiple drains are beneficial, how long drains can be left in place safely, and what volume of drainage is permissible for drain removal. A commonly used cut-off of 30 mL/day is used for discontinuation of drains in the USA [16, 17], but the threshold for drain removal is not well-defined or studied in the orthopedic oncology literature.

The present study was undertaken to determine whether certain aspects of surgical drain management might be related to infection for oncologic patients undergoing segmental bone resection. In particular, we were interested to know whether waiting until the rate of drain output was ≤ 30 mL/day before drain removal reduced the risk of infection. The study represents the largest cohort reported to date examining specifically the relationship of surgical drainage to periprosthetic infection in patients with modular endoprostheses.

## Methods

### Study design

A retrospective observational study with univariate and multivariate analysis was performed on a consecutive series of patients treated at one hospital meeting eligibility requirements. The primary endpoint was to identify predictive factors for periprosthetic infection. For the purposes of this study, the diagnosis of periprosthetic joint infection required a positive microbiologic culture from the joint fluid, surgical site, or wound. The diagnosis of infection was confirmed by supporting evidence from the patient’s history, physical examination, blood tests, and radiographs [18]. All cases of periprosthetic infection, no matter how late they occurred, were included in the analysis for risk factors. The study was performed with approval by the Institutional Review Board.

### Study population

**Fig. 1:**
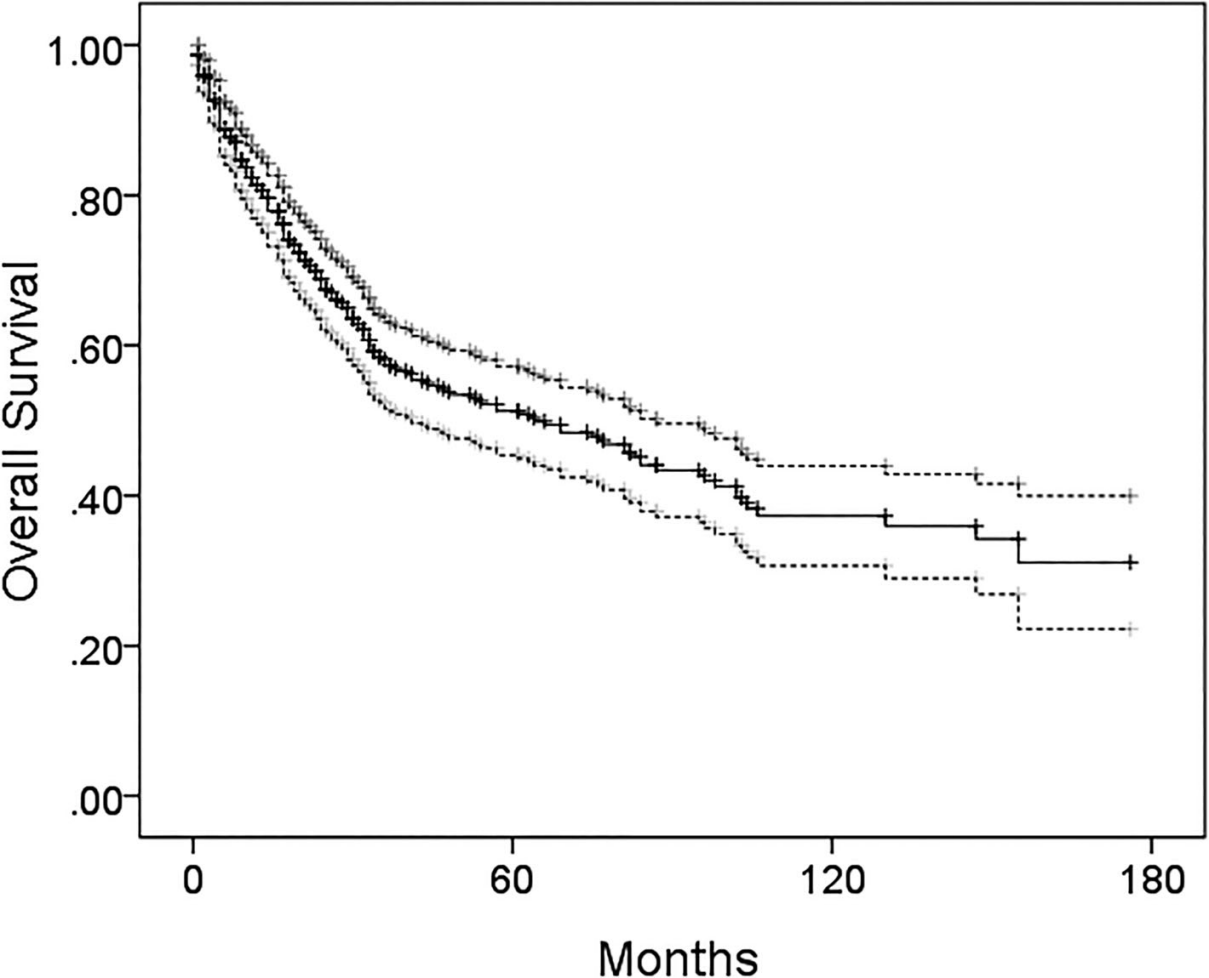
Overall survival. For the entire cohort of 295 patients, the overall survival was 52% at 5 years and 38% at 10 years by Kaplan-Meier survivorship analysis. The 95% confidence interval for overall survival is shown

The Orthopaedic Oncology Surgical Database at a single institution was queried for segmental bone resection and primary (non-revision) lower limb endoprosthetic reconstruction between 2000 and 2012. Minimum follow-up was 12 months unless patients died prior to 12 months. Out of 314 candidate cases, 19 (6%) were excluded for lack of follow-up and missing data. The remaining 295 patients formed the cohort for the present study. Demographic characteristics are listed in Table 1. The median follow-up was 36 months. Kaplan-Meier overall survival at 5 years for the study cohort was 52% (Fig. 1). There were 160 (54%) males and 135 (46%) females. The mean age was 41 years (range 6–84 years). Surgical sites were limited to proximal femur, distal femur, proximal tibia, and total femur. Disease categories included 14 (5%) benign tumors, 86 (29%) metastatic tumors (carcinomas and multiple myeloma), and 195 (66%) sarcomas.

**Table 1:** Demographic characteristics

Characteristic	Group	Value	***N***	%
Total patients			295	
Periprosthetic infection	Yes		31	11
	No		264	89
Follow-up (months)	Median	36		
	Range	1–201		
Vital status	Alive		131	44
	Dead		164	56
Gender	Male		160	54
	Female		135	46
Age (years)	Mean	41		
	Median	42		
	Range	6–84		
Diagnosis	Osteosarcoma		127	43
	Chondrosarcoma		30	10
	Ewing sarcoma		11	4
	Other sarcoma		27	9
	Giant cell tumor		14	5
	Renal cell carcinoma		31	11
	Other metastasic carcinomas and multiple myeloma		55	19
Site	Proximal femur		97	33
	Distal femur		131	44
	Total femur		13	4
	Proximal tibia		54	18
Chemotherapy	Preoperative		186	63
	Postoperative		173	59
	Any (pre- or post-)		206	70
	Both (pre- and post-)		152	52
	None		89	30
Radiation	Preoperative		38	13
	Postoperative		26	9
	None		231	78
Resection length (cm)	Mean	17		
	Median	16		
	Range	4–53		
Surgical drains	1		194	66
	2		90	31
	3		10	3
	4		1	0
Total drainage (L)	Mean	1.4		
	Median	1.1		
	Range	0.1–22.4		
Time of drainage (days)	Mean	8		
	Median	7		
	Range	1–48		
Average daily drainage^a^ (mL/day)	Mean	170		
	Median	157		
	Range	3–655		
Last day of drainage (mL)	Mean	72		
	Median	60		
	Range	0–510		
Operative time (hours)	Mean	5.3		
	Median	5.0		
	Range	1.7–14.7		
Estimated blood loss (mL)	Mean	809		
	Median	600		
	Range	50–9500		

^a^Average daily drainage = total drainage/time of drainage

*Abbreviations*: *L* liter

### Surgery and drain management

All patients had intravenous antibiotics administered immediately prior to skin incision. Patients received cefazolin for preoperative surgical prophylaxis unless patients had penicillin or cephalosporin allergy, in which case clindamycin was employed. For prolonged cases, the antibiotics were re-dosed after 6 h. There was no prospective protocol for drain management with regard to number of drains or duration of drains. The following information about postoperative drainage was collected from the patient files: number of drains, duration of drains, daily drain output, total drain output, mean output per day, and drain output on the last day (over 24 h).

### Statistics

Descriptive statistics were calculated and reported in terms of means, medians, and proportions. Bivariate linear regression was performed to assess the potential relationship of various continuous variables. Pearson’s correlation coefficient *R* was calculated for each pair of variables, and scatter plots with linear regression lines were generated by least squares. Univariate analysis for association with periprosthetic infection was performed for both categorical and continuous variables. The chi-square and Fisher’s exact tests were used to assess categorical variables in 2 × 2 comparisons. The means of continuous variables were compared with Student’s *t* test. The drain output on the last day was also analyzed as categorical variables by three different groupings with cut-off values at 30, 60, and 120 mL/day. Of note, 60 mL/day corresponded to the median drain output on the last day of the drain. The 120 mL/day value was intended to be a high value above median to test whether infections clustered in patients with very high output on the last day of the drain. Variables with *p* < .10 in univariate analysis were entered in multivariate Cox regression analysis for periprosthetic infection to identify independent risk factors. A forward conditional methodology based on likelihood ratio was used for entry of variables into the model. A *p* value < 0.05 was considered to be statistically significant. All analyses were performed with SPSS® version 24.0 for Windows (IBM Corp., Armonk, NY).

## Results

### Infection rate and pathologic organisms

Thirty-one of 295 cases (10.5%) developed periprosthetic infection at a median time of 13 months (range 1–108 months). Fifteen patients developed infection within a year of surgery; 11 patients developed infection between 1 and 5 years after surgery, and 5 patients developed infection after 5 years from surgery. The most common organisms isolated were *Staphylococcus aureus* (*n* = 12, of which 9 were methicillin-resistant), other *Staphylococcus* species (*n* = 6), *Streptococcus* species (*n* = 3), *Enterobacteriaceae* species (*n* = 3), and *Candida* species (*n* = 3). Rare organisms (one each) included *Propionibacterium acnes*, *Enterococcus*, *Stomatococcus*, *Corynebacterium*, *Prevotella*, *Fusobacterium*, and other anaerobic organisms (Table 2). Three patients had polymicrobial infections. Infections due to *Staphylococcus aureus* clustered in cases with acute onset within 1 year of surgery (Table 2).

**Table 2:** Pathogenic isolates and onset of infection

Organism	Onset of Infection (Number of cases)		
	< 1 year (number of cases)	1–5 years (number of cases)	> 5 years (number of cases)
*Staphylococcus aureus*, methicillin-resistant	6	2	1
*Staphylococcus aureus*, methicillin sensitive	3		
*Staphylococcus*, coagulase negative	2	1	
*Staphylococcus epidermidis*		1	1
*Staphylococcus warneri*			1
*Streptococcus,* Group B	1	1	
*Streptococcus,* Group G		1	
*Candida* species	1	1	1
*Enterococcus*	1		
*Stomatococcus*		1	
*Enterobacter*	1		
*Enterobacteriaceae* species		2	
*Propionibacterium acnes*			1
*Lactobacillus*		1	
*Corynebacterium*			1
*Gram variable rod* (not otherwise specified)		1	
*Fusobacterium*		1	
*Prevotella corporis*		1	

### Surgical drain output

**Fig. 2:**
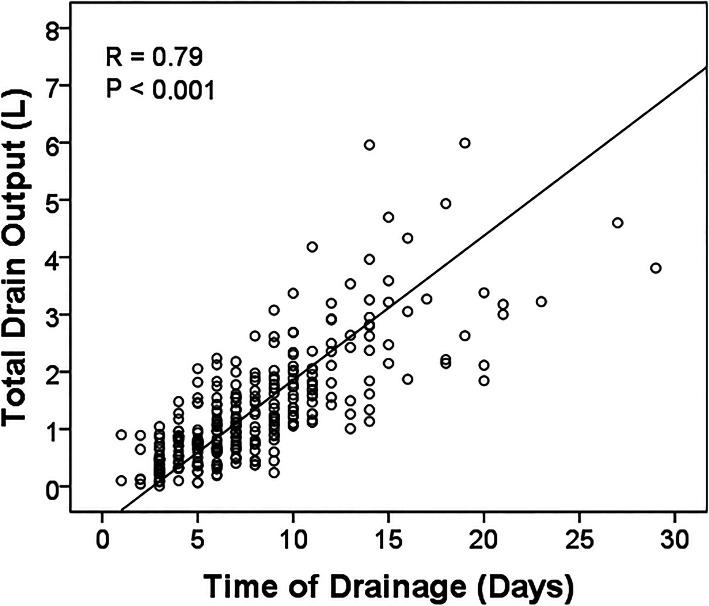
Drain output and duration of drains. Linear regression was performed to analyze the potential relationship between total drain output and duration of drains. A scatterplot with best-fit line is shown for total drain output (L, liters) and duration of surgical drains (Pearson’s correlation coefficient *R* = 0.79, *p* < 0.001)

**Fig. 3:**
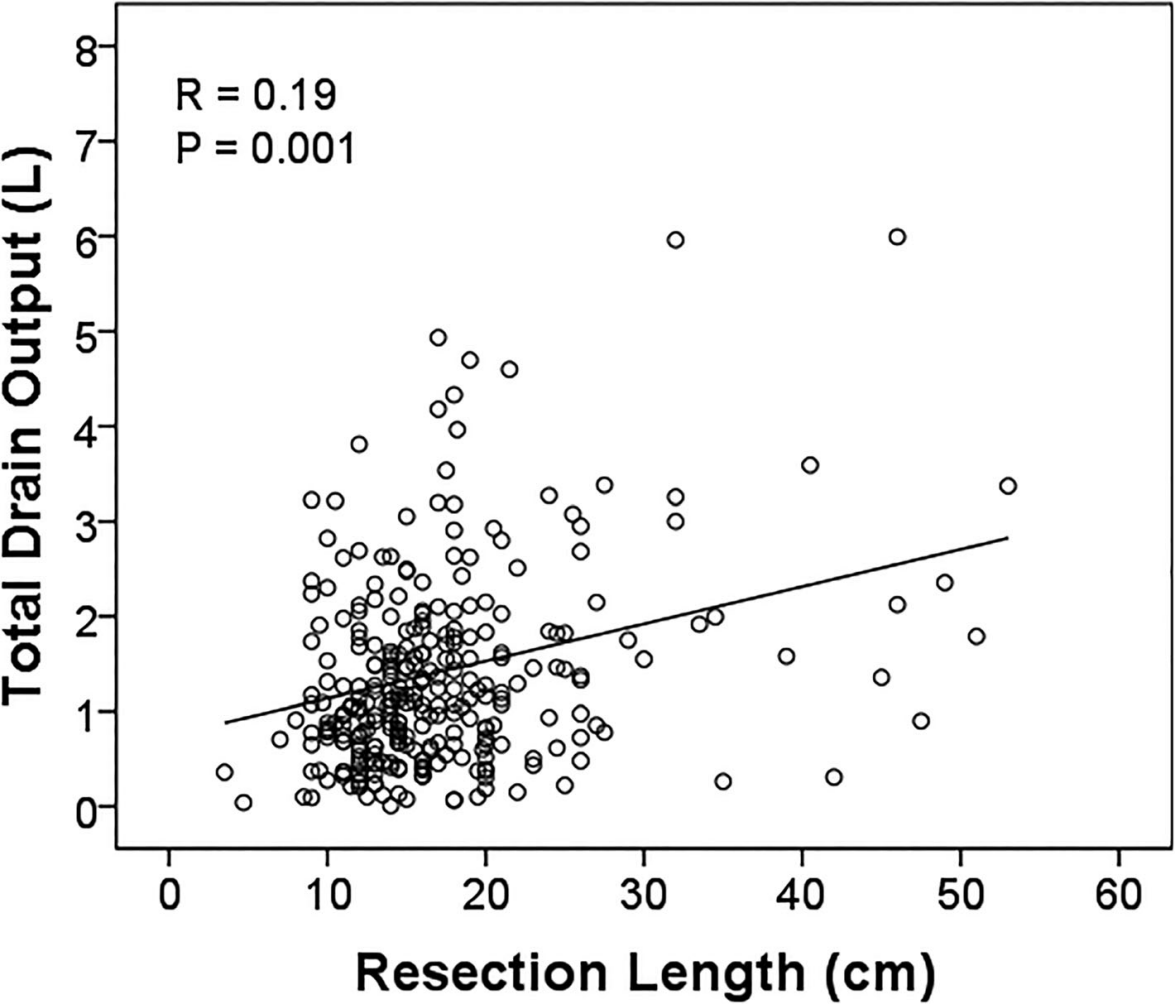
Total drain output and resection length. Linear regression was performed to analyze the potential relationship between total drain output and resection length. A scatterplot with best-fit line is shown for total drain output (L, liters) and resection length (Pearson’s correlation coefficient *R* = 0.19, *p* = 0.001)

The mean total amount of drain output was 1.4 L (range 0.1–22.4 L, standard deviation 1.6 L). Factors that correlated positively with total drain output included time of drainage (*R* = 0.79, *p* < 0.001, Fig. 2), mean drain output (*R* = 0.50, *p* < 0.001), volume of drainage on the last day (*R* = 0.47, *p* < 0.001), number of drains (*R* = 0.19, *p* = 0.001), resection length (*R* = 0.019, *p* = 0.001, Fig. 3), and age (*R* = 0.16, *p* = 0.007).

### Univariate analysis for periprosthetic infection

Individual factors were analyzed in univariate analyses for possible association with periprosthetic infection (Table 3). Of the non-drain-related variables, patients who had male gender, younger age, preoperative chemotherapy, postoperative chemotherapy, both pre- and postoperative chemotherapy, and sarcoma diagnosis were more likely to develop infection. Both the length of surgery and the need for re-dosing antibiotics during the surgery were associated with a greater rate of infection. Factors not associated with infection included length of resection, site of disease, preoperative radiation, postoperative radiation, and surgeon.

**Table 3:** Univariate analysis of factors for periprosthetic infection

Variable	Group or measure	Non-infected (***n*** = 264)	Infected (***n*** = 31)	***P*** value
Age (years)	Mean	42	35	0.11 ^a^
Resection length (cm)	Mean	17	20	0.05 ^a^
Gender	Male	135	25	0.002 ^b^
	Female	129	6	
Pathology	Benign	14	0	0.38 ^b^
	Sarcoma	170	25	0.08 ^b^
	Metastasis	80	6	0.30 ^b^
Site	Proximal femur	85	12	0.73 ^b^
	Distal femur	118	13	
	Proximal tibia	50	4	
	Total femur	11	2	
Surgeon	A	92	11	0.42 ^b^
	B	66	8	
	C	30	3	
	Others	76	9	
Preoperative chemotherapy	Yes	160	26	0.001 ^b^
	No	104	5	
Postoperative chemotherapy	Yes	85	22	0.01 ^b^
	No	170	4	
Both pre- and postoperative chemotherapy	Yes	127	25	0.001 ^b^
	No	137	6	
Any chemotherapy	Yes	180	26	0.10 ^b^
	No	84	5	
Preoperative radiotherapy	Yes	35	3	0.78 ^b^
	No	229	28	
Postoperative radiotherapy	Yes	24	2	1.00 ^b^
	No	240	29	
Number of drains	1	178	16	0.06 ^b^
	≥ 2	86	15	
Duration of drains (days)	Mean	8.1	10.0	0.04 ^a^
Total drainage (mL)	Mean	1,332	2,138	0.007 ^a^
Rate of drainage (mL/day)	Mean	167	198	0.07 ^a^
Last drainage^c^ (mL/day)	Mean	73	70	0.86 ^a^
Last drainage^c^ (mL/day)	≤ 30	78	10	0.84 ^b^
	> 30	186	21	
Last drainage^c^ (mL/day)	≤ 60	138	14	0.57 ^b^
	> 60	126	17	
Last drainage^c^ (mL/day)	≤ 120	214	27	0.62 ^b^
	> 120	50	4	
Duration of surgery (hours)	Mean	5.2	6.2	0.05 ^a^
Re-dose antibiotics in surgery	Yes	64	14	0.03 ^b^
	No	143	12	
Estimated blood loss (mL)	Mean	759	1,176	0.09 ^a^

^a^Student’s *t* test

^b^Pearson’s chi-square test or Fisher’s exact test

^c^Last day of drainage prior to drain removal

Of the drain-related variables, factors associated with infection included total volume of drainage, multiple (> 1) drains, days of drainage, and mean rate of drainage per day (Table 3). The amount of drainage on the last day did not predict infection (Table 3). The mean drainage on the last day was 70 mL/day for uninfected cases and 73 mL/day for infected cases (*p* = 0.94). Using the median rate of drainage on the last day (60 mL per day) as a cut-off value for grouping, 14 of 152 patients (9.2%) who had less than the median developed infections whereas 17 of 143 patients (11.9%) with greater than the median had infections (*p* = 0.57). Using the cut-off of 30 mL per day on the last day of the drain, patients who had less than 30 mL/day did not have significantly fewer infections than patients who had greater than 30 mL/day (Table 3). Similarly, using a cut-off value of 120 mL/day did not reveal a significant association with infection (Table 3).

### Multivariate analysis

The following factors with a p value < 0.10 in univariate analysis were entered into a multivariate Cox regression analysis to identify independent covariates of infection: gender, resection length, preoperative chemotherapy, postoperative chemotherapy, any chemotherapy, both pre- and postoperative chemotherapy, number of drains, drain duration, rate of drainage, total volume of output, operative time, estimated blood loss, re-dosing of antibiotics, and sarcoma diagnosis. The independent factors in the final equation included sarcoma diagnosis (HR 4.13, 95% CI 1.4–12.2, *p* = 0.01) and preoperative chemotherapy (HR 3.29, 95% CI 1.1–9.6, *p* = 0.03) (Table 4). Male diagnosis did not reach statistical significance (HR 2.5, 95% CI 0.9–6.8, *p* = 0.08)

**Table 4:** Multivariate Cox regression analysis for periprosthetic infection–factors in final equation

Variable	B	SE	HR (ℯ^**B**^)	95% CI	***P*** value
Male gender	0.92	0.51	2.50	0.9–6.8	0.08
Chemotherapy (preoperative)	1.19	0.55	3.29	1.1–9.6	0.03
Sarcoma	1.42	0.55	4.13	1.4–12.2	0.01

*B* Beta coefficient; *SE* standard error; *HR* hazard ratio; *CI* confidence interval

## Discussion

Infection is a major cause of failure after segmental bony resection and modular endoprosthetic reconstruction. The rate of periprosthetic infection in this study was 10.5%, which is similar to the rate reported in other articles for modular endoprostheses [1, 19]. The risk factors for infection can be broadly placed into two main categories: patient-related and treatment-related variables [20–22]. The surgical drain is a treatment-related risk factor, but it has not received much attention in the literature pertaining to segmental endoprostheses. Previous studies have examined the relationship between infection and drainage in conventional arthroplasty [13, 23]. Prolonged duration of an indwelling surgical drain increases the likelihood of infection [13, 24]. Each additional day of having a surgical drain increases the risk of wound infection by 42% for hip arthroplasty and 29% for knee arthroplasty [13]. It has also been shown that the degree of bacteriological contamination in drainage tubes increases after 3 days [24]. On the other hand, surgeons may fear removing a drain too early could lead to a large hematoma, which might also be a risk factor for infection [25].

A practical question is when to remove the drain. Although many surgeons wait until the rate of drainage diminishes to 30 mL/day, there is no published data to justify this practice. In our study, the mean rate of output at the time of drain removal was 72 mL/day, and there was no statistical difference in infected and uninfected cases. Furthermore, using the median value of 60 mL/day, there was no difference in infection rate for patients below or above the median. This was also true for a cut-off value of 30 mL/day. Our data suggests that one does not necessarily need to wait until the drainage reaches 30 mL/day to remove the drain. In fact, waiting excessively long for the drain output to reach this level could be detrimental, and our univariate analysis shows an association between duration of drains and risk of infection, consistent with data from conventional arthroplasty [13, 23]. Some surgeons have advocated using no surgical drains at all, since data from randomized controlled trials for conventional arthroplasty do not support the routine use of closed suction drains [25, 26]. Whether this concept can be applied to oncologic cases that involve extensive dissection remains to be determined.

High volume of drain output has been reported to be an adverse finding for infection risk [13]. In our study, it was a significant factor in univariate but not multivariate analysis. There are several potential reasons for this. The sample size may have been inadequate to establish high drain output as an independent variable. In addition, high drain output may have been dependent upon other variables. In our analysis, total drain output correlated with a number of factors, the most significant being duration of the surgical drains (*R* = 0.79). Total drain output also had a significant correlation with multiple drains, but the degree of correlation was weak (*R* = 0.19). A variety of other factors could also contribute to the total drain output including coagulopathy, hemostasis at the time of closure, wound closure technique, nutritional status, and anti-coagulant medications, especially for prophylaxis against venous thromboembolism [13]. We did not attempt to analyze the effect of these factors on drainage in this study.

Some surgeons believe that early infection is related to wound problems, while late infection is due to hematogenous spread [27]. It may be difficult to decide what time point should be the cut-off for early infections. Due to the possibility that seeding of an implant in the perioperative period could manifest in a delayed manner, particularly for indolent organisms, we chose to include all infections in our analysis. Indeed, at least 6 of the late infections in our series were due to organisms such as *Propionibacterium acnes*, which might be deemed indolent. In contrast, *Staphylococcus aureus*, particularly the more virulent methicillin-resistant type, clustered in the early infections with onset within 1 year of surgery.

The multivariate analysis indicated that sarcoma diagnosis and preoperative chemotherapy were independent predictive factors for infection. We had included both factors in the analysis since we could not determine a priori which would potentially be a stronger predictive factor. Furthermore, even though these two factors might seem similar, they are not equivalent, for some sarcoma patients, such as those with conventional chondrosarcoma, do not receive preoperative chemotherapy. What this finding might suggest is that more extensive resections, which would include bone and soft tissue, may play an important role in the development of infection. In contrast, resections of aggressive benign tumors, such as giant cell tumor of bone, usually do not require substantial soft tissue removal. This is also generally true for many metastatic carcinomas that are treated with resection and endoprosthetic replacement.

Limitations of this study include the retrospective nature of the analysis and the fact that management of drains was not determined by a prospective protocol. We chose to focus our study on aspects of drain management. There may be other factors important to infection, such as nutritional status of patients and comorbidities, so our list of risk factors should not be considered a complete, exhaustive list. The rate of infection may be an underestimate of the true rate since patients who are lost to follow-up may still be at risk for subsequent infection.

## Conclusions

Our data does not support waiting until drainage reaches 30 mL/day before drain removal. There was no difference in infection rate for patients who had drain removal less than or greater than 30 mL/day. In this cohort, the mean drain output at the time of removal was 72 mL/day, and there was no apparent increase in risk at this level of output either. Sarcoma diagnosis and preoperative chemotherapy were independent factors for infection in multivariate analysis. Future work is needed to examine the predictive value of other variables in periprosthetic infection and whether some patients may be managed without drains altogether.

## Data Availability

The datasets used and/or analyzed during the current study are available from the corresponding author on reasonable request.
